# The impact of preoperative anticoagulant administration on postoperative coagulation function and blood loss following posterior lumbar interbody fusion: a case–control study

**DOI:** 10.3389/fmed.2026.1753809

**Published:** 2026-03-09

**Authors:** Hongqi Zhan, Shenshen Hao, Shuaizhi Li, Xiaoya Zhu, Shengli Dong, Shuai Liu, Hongke Li, Ting Wang

**Affiliations:** 1 Affiliated Hospital of Qingdao University, Qingdao, Shandong, China; 2 Department of Spine, Affiliated Hospital of Qingdao University, Qingdao, Shandong, China; 3 Department of Spine and Bone Oncology, General Hospital of Pingmei Shenma Medical Group, Pingdingshan, Henan, China; 4 Department of Bone Disease Area of Limbs, General Hospital of Pingmei Shenma Medical Group, Pingdingshan, Henan, China; 5 Interventional Operating Room, Pingdingshan the Second People’s Hospital, Pingdingshan, Henan, China

**Keywords:** anticoagulant drugs, blood loss, coagulation function, indobufen, low-molecular-weight heparin, posterior lumbar interbody fusion

## Abstract

**Background:**

Currently, there are few reports on the impact of preoperative anticoagulant administration on perioperative coagulation function and blood loss. Therefore, this study observed the effects of preoperative anticoagulant administration on postoperative coagulation function and blood loss in posterior lumbar interbody fusion (PLIF).

**Methods:**

A retrospective analysis was conducted on the data of 61 patients who underwent PLIF from October 2021 to March 2023. Patients who used anticoagulants [low-molecular-weight heparin (LMWH) or indobufen] preoperatively were recorded as the observation group (*n* = 29), and those who did not were recorded as the control group (*n* = 32). The observation group was further divided into two subgroups based on the type of anticoagulant, namely the LMWH group (*n* = 11) and the indobufen group (*n* = 18). The observation indicators included surgical time, intraoperative blood loss, postoperative drainage volume, number of blood transfusions, incision healing, deep vein thrombosis (DVT) of the lower limbs, postoperative activated partial thrombin time (APTT), prothrombin time (PT), thrombin time (TT), fibrinogen (FIB), platelet (PLT), postoperative hospital stay, and hemoglobin (HB), red blood cells (RBC), and hematocrit (HCT) on the postoperative 1st, 4th, 7th, and last-tested days.

**Results:**

All surgeries completed successfully with incisions healed in grade A, without DVT occurred. There were no significant differences in surgical time, intraoperative blood loss, postoperative drainage volume, number of blood transfusions, and postoperative hospital stay between the two groups (*p* > 0.05). There were no significant differences in postoperative TT, FIB, APTT, and PLT between the two groups (*p* > 0.05). However, there was a significant difference in postoperative PT between the two groups (*p* < 0.05). There were no significant differences in the above-mentioned indicators between the two subgroups (*p* > 0.05). Multivariate regression analysis indicated that the surgical segment was an independent influencing factor for intraoperative blood loss (*p* < 0.05). There were no significant differences in HB, RBC, and HCT between the two groups on the postoperative 1st, 4th, 7th, and last-tested days (*p* > 0.05).

**Conclusion:**

The use of anticoagulants before PLIF has no significant effect on postoperative TT, FIB, APTT, and PLT (except for PT), and does not significantly affect blood loss.

## 1 Background

Lumbar degenerative diseases (LDD), such as lumbar disc herniation (LDH), lumbar spinal stenosis (LSS), and lumbar spondylolisthesis (LS), are common diseases that can significantly reduce patients’ quality of life. With the popularization of surgical concepts, an increasing number of patients choose surgical treatment when they are dissatisfied with conservative treatment (1). Surgery can effectively relieve symptoms and improve patients’ quality of life (2). Posterior lumbar interbody fusion (PLIF) has become one of the commonly used surgical modalities for treating LDD. However, it is a complex surgical procedure involving multiple steps, requiring a long operation time and potentially leading to substantial blood loss (3–6). Excessive bleeding may cause anemia, often requiring blood transfusion for correction. Blood transfusion not only exacerbates the blood shortage but also carries the risk of transfusion-related diseases and adverse events, such as local allergic reactions, systemic allergic reactions, and transfusion-related acute lung injury.

PLIF is also a high-risk factor for thrombosis formation, anticoagulant drugs for preventing thrombosis have been widely accepted (7–9). However, there is no consensus on when to use anticoagulant drugs. Theoretically, the use of anticoagulant drugs before surgery may increase perioperative bleeding. Therefore, anticoagulant drugs are not routinely used before surgery (10). However, in clinical practice, there are often patients who need anticoagulant drugs before surgery. They include patients with hypertension who use preventive medication to reduce the risk of thrombosis and patients who have undergone heart surgery and need long-term anticoagulant drugs to prevent restenosis or occlusion of blood vessels. For such patients, aspirin is commonly used as an anticoagulant drug. It is an antiplatelet drug that mainly exerts its antithrombotic effect by inhibiting platelet (PLT) aggregation (11). However, such drugs may increase the risk of perioperative bleeding, so alternative drugs are usually selected for bridging anticoagulation therapy before surgery.

Among the alternative drugs, low-molecular-weight heparin (LMWH) is the most commonly used (12). It has the advantages of high bioavailability, long half-life, and relatively low bleeding risk (13). However, it requires subcutaneous injection, which may be difficult for some patients to accept. They can choose indobufen, an oral formulation, as an alternative anticoagulant drug. As a new-generation antiplatelet drug, indobufen can effectively inhibit the activity of platelet cyclooxygenase-1, and its biochemical, functional and clinical effects are similar to those of standard-dose aspirin (14). In addition, our previous study found that using indobufen tablets as an alternative anticoagulant treatment before double-segment PLIF is feasible (15).

However, there are relatively few studies on the use of anticoagulants before PLIF. Therefore, we conducted this study to investigate the effects of anticoagulant drugs before PLIF on postoperative coagulation function and blood loss.

## 2 Materials and methods

### 2.1 Study design

This study was a single-center retrospective case–control study. The data collection period was from October 2021 to March 2023. A total of 61 patients with LDD who underwent surgical treatment and met the inclusion and exclusion criteria were included in this study. Among the enrolled patients, 26 were male and 35 were female. The age range was 38 to 81 years, with an average age of (63.72 ± 8.74) years. Patients were divided into the observation group (*n* = 29) and the control group (*n* = 32) based on whether they used anticoagulant drugs (LMWH or indobufen tablets) before surgery. Since the observation group used two anticoagulant drugs, it was further divided into two subgroups: 11 cases in the LMWH subgroup and 18 cases in the indobufen subgroup. The inclusion criteria were as follows: (a) Patients who underwent PLIF surgery. (b) The surgical segments included 1 to 4 segments. (c) Patients underwent lumbar surgery for the first time. (d) The American Society of Anesthesiologists physical status classification system was classified as grade II. (e) All surgeries were performed by the same surgical team. The exclusion criteria were as follows: (a) The patient had hematological disorders prior to surgery. (b) Preoperative lower extremity deep vein thrombosis (DVT) was present in the patient. (c) Patients with diabetes before surgery. (d) Patients who used antifibrinolytic hemostatic drugs, such as tranexamic acid (16), during the perioperative period. (e) Intraoperative or postoperative cerebrospinal fluid leakage occurred in the patient.

The preoperative general data included gender, age, body mass index (BMI), disease type, surgical segment, coexisting hypertension, activated partial thromboplastin time (APTT), prothrombin time (PT), thrombin time (TT), fibrinogen (FIB), PLT, hemoglobin (HB), red blood cell (RBC), hematocrit (HCT), C-reactive protein (CRP), and erythrocyte sedimentation rate (ESR).

### 2.2 Research methods

The observation group received alternative anticoagulant therapy with LMWH or indobufen tablets (produced by Hangzhou Zhongmei Huadong Pharmaceutical Co., Ltd., China, with batch number H20194067) within 1 week before the operation. LMWH was administered subcutaneously at a dose of 4,250 IU once daily, and indobufen tablets were taken orally twice a day, 10 mg each time, for 7 days before surgery. Anticoagulants were discontinued 1 day preoperatively and resumed on postoperative first day. The control group did not received anticoagulant drugs. Both groups underwent standard PLIF under general anesthesia in the prone position with the same surgical procedures. Two negative pressure drainage tubes were placed during the operation. Similar routine treatments were received after surgery. The drainage tubes were removed when the drainage volume was less than 50 mL/24 h. The recovery of the surgical incision and the formation of DVT in the lower limbs were observed. During the period of bed rest, ankle pump function and quadriceps contraction function exercises were performed. On the first day after surgery, rehabilitation activities could be carried out under the protection of a lumbar brace.

Blood transfusion standards were based on the following three criteria (17). Firstly, blood transfusion was not required when the HB level exceeds 100 g/L. Secondly, blood transfusion was required when the HB level was below 70 g/L. Thirdly, the decision to transfuse blood was jointly evaluated by the surgeon and the anesthesiologist when the HB level was between 70 g/L and 100 g/L.

### 2.3 Data collection

Observation indicators included blood loss-related indicators, coagulation-related indicators, anemia-related indicators, surgical time, incision healing, DVT of the lower limbs, and postoperative hospital stay. Blood loss-related indicators included intraoperative blood loss, the number of blood transfusions, and postoperative drainage volume. Coagulation-related indicators included postoperative APTT, PT, TT, FIB, and PLT. Anemia-related indicators included HB, RBC, and HCT on the postoperative 1st, 4th, 7th, and last-tested days.

Coagulation-related indicators were all collected from venous blood samples taken from patients in the morning of postoperative 1st day before infusion. Similarly, the anemia-related indicators was measured on the days. Intraoperative blood loss was calculated using the formula: Intraoperative blood loss = Volume of blood collected by the suction device + Blood volume adsorbed on gauze − Volume of intraoperative irrigation fluid. The volume of blood absorbed on gauze during the operation was equal to the net increase in the weight of the gauze, that was, the total weight of the gauze after surgery minus the net weight of the gauze before surgery. A 1 g net weight gain for the gauze was converted to 1 mL of blood loss. To monitor the occurrence of DVT after surgery, the condition of the lower limbs of the patients was observed every day, including the circumference of the lower limbs, pain, and skin temperature. Generally, when the patients presented with inconsistent circumferences of the lower limbs, sudden pain, or changes in skin temperature, an urgent lower limb vascular ultrasound examination was performed to determine whether DVT existed.

### 2.4 Statistical analysis

Data analysis was conducted using SPSS statistical software (version 27.0). Normality tests were performed on continuous variables. Variables following a normal distribution were expressed as mean ± standard deviation, and independent samples t-test was employed for the comparison between the two groups. Non-normally distributed data were presented as M [P25, P75] and Mann–Whitney *U* test was employed for the comparison between the two groups. The chi-square test was employed for the comparison of categorical data between groups. The rank sum test was employed to compare the surgical segments between the two groups. The repeated-measures analysis of variance was used to compare the differences in HB, RBC, and CRP between the two groups at different time points. If Mauchly’s sphericity test was not satisfied, Greenhouse–Geisser correction was applied.

To mitigate potential bias and adjust for potential confounding factors, further multiple linear or logistic regression analyses were performed. Blood loss-related indicators were used as dependent variables, including intraoperative blood loss, blood transfusion, postoperative drainage volume, RBC, HB, and HCT on the postoperative 1st day. Variables that might affect blood loss-related indicators were used as independent variables, including anticoagulant drugs, coexisting hypertension, disease type, and surgical segments.

## 3 Result

### 3.1 Comparison of preoperative general data between the two groups

There were no significant difference in preoperative general data between the two groups (*p* > 0.05), including gender, age, BMI, disease type, surgical segment, coexisting hypertension, APTT, PT, TT, FIB, PLT, HB, RBC, HCT, CRP and ESR. The detailed data were shown in Table 1.

**Table 1:** Comparison of the general data of the two groups.

Groups	Observation group (*n* = 29)	Control group (*n* = 32)	*t*/*χ*^2^/*Z* value	*p*-value
Age, year	65.34 ± 8.47	62.25 ± 8.85	1.392	0.169
Gender, *n*			0.035	0.852
Male	12	14		
Female	17	18		
BMI, kg/m^2^	25.322 ± 3.120	25.111 ± 3.436	0.250	0.803
Disease type, *n*			0.774	0.679
LDH	5	5		
LSS	17	16		
LS	7	11		
Surgical segment, *n*			−1.354	0.176
One	13	21		
Two	11	6		
Three	4	4		
Four	1	1		
Comorbid hypertension, *n*			1.184	0.277
Yes	12	9		
No	17	23		
HB, g/L	136.069 ± 14.375	136.438 ± 10.163	−0.116	0.908
RBC, 10^12^/L	4.364 ± 0.416	4.292 ± 0.298	0.785	0.436
HCT, L/L	0.406 ± 0.040	0.399 ± 0.031	0.718	0.476
APTT, s	30.803 ± 3.347	31.519 ± 2.708	−0.921	0.361
PT, s	10.876 ± 0.750	11.184 ± 0.799	−1.551	0.126
TT, s	14.979 ± 1.086	14.925 ± 0.813	0.222	0.825
FIB, g/L	2.941 ± 0.456	2.893 ± 0.414	0.431	0.668
PLT, 10^9^/L	228.655 ± 65.516	211.531 ± 47.559	1.158	0.252
CRP, mg/L	0.21 [0; 1.35]	0.70 [0; 2.06]	−1.103	0.270
ESR, mm/h	16 [9; 24]	15 [9; 25]	−0.434	0.665

### 3.2 Comparison of preoperative general data between the two subgroups

There were no significant difference in preoperative general data between the two subgroups (*p* > 0.05), including gender, age, BMI, disease type, surgical segment, coexisting hypertension, APTT, PT, TT, FIB, PLT, HB, RBC, HCT, CRP and ESR. The detailed data were shown in Table 2.

**Table 2:** Comparison of the general data of the two subgroups.

Groups	Indobufen group (*n* = 18)	LMWH group (*n* = 11)	*t*/*χ*^2^/*Z* value	*p*-value
Age, year	64.78 ± 8.92	66.27 ± 8.01	−0.455	0.653
Gender, *n*			0.121	0.728
Male	7	5		
Female	11	6		
BMI, kg/m^2^	24.563 ± 3.150	26.564 ± 2.764	−1.736	0.094
Disease type, *n*			1.545	0.462
LDH	4	1		
LSS	9	8		
LS	5	2		
Surgical segment, *n*			−0.778	0.436
One	8	8		
Two	5	6		
Three	4	0		
Four	1	0		
Comorbid hypertension, *n*			0.121	0.728
Yes	7	5		
No	11	6		
HB, g/L	136.670 ± 14.605	135.090 ± 14.639	0.282	0.780
RBC, 10^12^/L	4.401 ± 0.479	4.303 ± 0.297	0.605	0.550
HCT, L/L	0.412 ± 0.041	0.396 ± 0.036	1.010	0.321
APTT, s	30.794 ± 3.618	30.818 ± 3.019	−0.018	0.986
PT, s	10.717 ± 0.757	11.136 ± 0.692	−1.495	0.147
TT, s	15.101 ± 0.896	14.779 ± 1.366	0.696	0.497
FIB, g/L	2.896 ± 0.488	3.016 ± 0.410	−0.685	0.499
PLT, 10^9^/L	232.110 ± 66.949	223.000 ± 65.897	−0.685	0.499
CRP, mg/L	0 [0; 1.420]	0.63 [0; 1.420]	−0.620	0.535
ESR, mm/h	14 [7; 24]	17 [12; 26]	−0.945	0.345

### 3.3 Multivariate linear regression analysis with intraoperative blood loss as the dependent variable

Multivariate linear regression analysis was conducted with intraoperative blood loss as the dependent variable and anticoagulant drugs, coexisting hypertension, disease type, and surgical segment as independent variables. The results showed that the surgical segment was a factor influencing intraoperative blood loss, while anticoagulant drugs, coexisting hypertension and disease type were not. The detailed data were shown in Table 3.

**Table 3:** Multivariate linear regression analysis with intraoperative blood loss as the dependent variable.

Variables	*B*	SE	*t* value	*p*-value	95% CI	
					Lower limit	Upper limit
Constant	1397.700	184.109	7.592	0.000	1028.424	1766.977
Anticoagulant drugs (1 = yes, 0 = no)	−78.700	53.705	−1.465	0.149	−186.419	29.019
Coexisting hypertension (1 = yes, 0 = no)	−46.453	55.474	−0.837	0.406	−157.719	64.812
Disease type (reference: LDH)						
LSS	43.576	73.070	0.596	0.553	−102.983	190.136
LS	102.485	79.837	1.284	0.205	−57.648	262.619
Surgical segment (reference: four)	−0.312	0.261	−1.196	0.235	−0.830	0.206
One	−953.163	148.281	−6.428	0.000	−1250.577	−655.749
Two	−706.196	151.757	−4.653	0.000	−1010.582	−401.810
Three	−423.121	162.832	−2.599	0.012	−749.720	−96.522

### 3.4 Multivariate logistic regression analysis with blood transfusion as the dependent variable

Multivariate logistic regression analysis was conducted with blood transfusion as the dependent variable and anticoagulant drugs, coexisting hypertension, disease type and surgical segment as independent variables. The results showed that the surgical segment was a factor influencing blood transfusion, while anticoagulant drugs, coexisting hypertension and disease type were not. The detailed data were shown in Table 4.

**Table 4:** Multivariate logistic regression analysis with blood transfusion as the dependent variable.

Variables	*B*	SE	Wald	*p*-value	OR	95% CI	
						Lower limit	Upper limit
Constant	−26.208	57585.999	0.000	1.000			
Anticoagulant drugs (1 = yes, 0 = no)	−0.608	0.922	0.434	0.510	0.545	0.089	3.321
Coexisting hypertension (1 = yes, 0 = no)	1.675	1.210	1.917	0.166	5.340	0.498	57.219
Disease type (reference: LDH)							
LSS	−0.511	1.337	0.146	0.702	0.600	0.044	8.238
LS	0.446	1.064	0.176	0.675	1.562	0.0194	12.568
Surgical segment (reference: four)							
One	−24.553	27990.696	0.000	0.999	0.000	0.000	—
Two	−22.440	27990.696	0.000	0.999	0.000	0.000	—
Three	−0.339	31075.856	0.000	1.000	0.712	0.000	—

### 3.5 Multivariate linear regression analysis with postoperative drainage volume as the dependent variable

Multivariate linear regression analysis was conducted with postoperative drainage volume as the dependent variable and potential factors influencing blood loss-related indicators as independent variables, including anticoagulant drugs, coexisting hypertension, disease type and surgical segment. The results showed that the surgical segment was a factor influencing postoperative drainage volume, while anticoagulant drugs, coexisting hypertension and disease type were not. The detailed data were shown in Table 5.

**Table 5:** Multivariate linear regression analysis with postoperative drainage volume as the dependent variable.

Variables	*B*	SE	*t* value	*p*-value	95% CI	
					Lower limit	Upper limit
Constant	582.408	101.187	5.756	0.000	379.452	785.363
Anticoagulant drugs (1 = yes, 0 = no)	−31.163	29.517	−1.056	0.296	−90.366	28.040
Coexisting hypertension (1 = yes, 0 = no)	−8.374	30.488	−0.275	0.785	−69.526	52.778
Disease type (reference: LDH)						
LSS	−6.476	40.159	−0.161	0.872	−87.026	74.073
LS	24.929	43.879	0.568	0.572	−63.081	112.938
Surgical segment (reference: four)	−0.312	0.261	−1.196	0.235	−0.830	0.206
One	−237.152	81.496	−2.910	0.005	−400.612	−73.692
Two	−168.874	83.406	−2.025	0.048	−336.166	−1.582
Three	−128.606	89.493	−1.437	0.157	−308.106	50.894

### 3.6 Multivariate linear regression analysis with HB on the postoperative 1st day as the dependent variable

Taking HB on the postoperative 1st day as the dependent variable and the factors that might affect the blood loss-related indicators as the independent variables, including anticoagulant drugs, coexisting hypertension, disease type and surgical segment, a multivariate linear regression analysis was conducted. The results showed that anticoagulant drugs, coexisting hypertension, disease type, and surgical segment were not the influencing factors of HB on the postoperative 1st day. The detailed data were shown in Table 6.

**Table 6:** Multivariate linear regression analysis with HB on the postoperative 1st day as the dependent variable.

Variables	*B*	SE	*t* value	*p*-value	95% CI	
					Lower limit	Upper limit
Constant	111.139	10.757	10.332	0.000	89.563	132.715
Anticoagulant drugs (1 = yes, 0 = no)	−4.012	3.138	−1.279	0.207	−10.306	2.281
Coexisting hypertension (1 = yes, 0 = no)	−1.668	3.241	−0.515	0.609	−8.169	4.833
Disease type (reference: LDH)						
LSS	2.714	4.269	0.636	0.528	−5.849	11.277
LS	3.164	4.665	0.678	0.501	−6.193	12.520
Surgical segment (reference: four)	−0.312	0.261	−1.196	0.235	−0.830	0.206
One	10.256	8.664	1.184	0.242	−7.121	27.634
Two	4.996	8.867	0.563	0.576	−12.789	22.780
Three	15.947	9.514	1.676	0.100	−3.135	35.030

### 3.7 Multivariate linear regression analysis with RBC on the postoperative 1st day as the dependent variable

Taking RBC on the postoperative 1st day as the dependent variable and the factors that might affect the blood loss-related indicators as the independent variables, including anticoagulant drugs, coexisting hypertension, disease type and surgical segment, a multivariate linear regression analysis was conducted. The results showed that anticoagulant drugs, coexisting hypertension, disease type, and surgical segment were not the influencing factors of RBC on the postoperative 1st day. The detailed data were shown in Table 7.

**Table 7:** Multivariate linear regression analysis with RBC on the postoperative 1st day as the dependent variable.

Variables	*B*	SE	*t* value	*p*-value	95% CI	
					Lower limit	Upper limit
Constant	3.677	0.328	11.210	0.000	3.019	4.335
Anticoagulant drugs (1 = yes, 0 = no)	−0.158	0.096	−1.649	0.105	−0.350	0.034
Coexisting hypertension (1 = yes, 0 = no)	−0.053	0.099	−0.532	0.597	−0.251	0.146
Disease type (reference: LDH)						
LSS	−0.019	0.130	−0.145	0.885	−0.280	0.242
LS	0.023	0.142	0.161	0.872	−0.262	0.308
Surgical segment (reference: four)	−0.312	0.261	−1.196	0.235	−0.830	0.206
One	0.288	0.264	1.089	0.281	−0.242	0.817
Two	0.173	0.270	0.641	0.525	−0.369	0.715
Three	0.374	0.290	1.288	0.203	−0.208	0.955

### 3.8 Multivariate linear regression analysis with HCT on the postoperative 1st day as the dependent variable

Taking HCT on the postoperative 1st day as the dependent variable and the factors that might affect blood loss-related indicators as independent variables, including anticoagulant drugs, coexisting hypertension, disease type and surgical segment, a multivariate linear regression analysis was conducted. The results showed that anticoagulant drugs, coexisting hypertension, disease type, and surgical segment were not the influencing factors of HCT on the postoperative 1st day. The detailed data were shown in Table 8.

**Table 8:** Multivariate linear regression analysis with HCT on the postoperative 1st day as the dependent variable.

Variables	*B*	SE	*t* value	*p*-value	95% CI	
					Lower limit	Upper limit
Constant	0.334	0.032	10.479	0.000	0.270	0.398
Anticoagulant drugs (1 = yes, 0 = no)	−0.012	0.009	−1.299	0.200	−0.031	0.007
Coexisting hypertension (1 = yes, 0 = no)	−0.004	0.010	−0.414	0.681	−0.023	0.015
Disease type (reference: LDH)						
LSS	0.001	0.013	0.063	0.950	−0.025	0.026
LS	−0.001	0.014	−0.042	0.967	−0.028	0.027
Surgical segment (reference: four)	−0.312	0.261	−1.196	0.235	−0.830	0.206
One	0.028	0.026	1.090	0.281	−0.024	0.080
Two	0.015	0.026	0.553	0.582	−0.038	0.067
Three	0.042	0.028	1.499	0.140	−0.014	0.099

### 3.9 Comparison of surgery and blood loss-related indicators between the two groups

All patients successfully completed the surgery, and all incisions healed at grade A. No lower limb DVT occurred during the observation period. There was no statistically significant difference in surgical time and postoperative hospital stay between the two groups (*p* > 0.05). Regarding the blood loss-related indicators, there were no statistically significant differences in the intraoperative blood loss, postoperative drainage volume, and the number of blood transfusions between the two groups (*p* > 0.05). The detailed data were shown in Table 9.

**Table 9:** Comparison of surgery and blood loss-related indicators between the two groups.

Groups	Observation group (*n* = 29)	Control group (*n* = 32)	*t*/*χ*^2^ value	*p*-value
Surgical time, min	194.138 ± 49.842	173.813 ± 58.089	1.459	0.150
Intraoperative blood loss, mL	500 [300; 800]	400 [300; 600]	−1.539	0.124
Postoperative drainage volume, mL	350 [310; 400]	305 [225; 360]	−1.844	0.065
Blood transfusion, *n*			0.658	0.417
Yes	10	8		
No	19	24		
Postoperative hospital stay, day	12 [10; 16]	14 [9; 17]	−0.754	0.451

### 3.10 Comparison of surgery and blood loss-related indicators between the two subgroups

There were no statistically significant differences in surgical time, intraoperative blood loss, postoperative drainage volume, the number of blood transfusions, and postoperative hospital stay between the two subgroups (*p* > 0.05). The detailed data were shown in Table 10.

**Table 10:** Comparison of surgery and blood loss-related indicators between the two subgroups.

Groups	Indobufen group (*n* = 18)	LMWH group (*n* = 11)	*t*/*χ*^2^ value	*p*-value
Surgical time, min	192.610 ± 45.952	196.640 ± 57.925	−0.207	0.837
Intraoperative blood loss, mL	627.780 ± 359.420	545.450 ± 284.125	0.645	0.524
Postoperative drainage volume, mL	340 [308; 400]	360 [300; 410]	−0.293	0.770
Blood transfusion, *n*			0.028	0.868
Yes	6	4		
No	12	7		

### 3.11 Comparison of coagulation-related indicators between the two groups

There were no significant differences in postoperative TT, FIB, APTT, and PLT between the two groups (*p* > 0.05). There was a statistically significant difference in postoperative PT between the two groups (*p* < 0.05). The detailed data were shown in Table 11.

**Table 11:** Comparison of the postoperative coagulation-related indicators between the two groups.

Groups	Observation group (*n* = 29)	Control group (*n* = 32)	*t* value	*p*-value
APTT, s	28.207 ± 2.276	28.778 ± 2.433	−0.944	0.349
PT, s	11.676 ± 0.934	12.369 ± 0.997	−2.792	0.007
TT, s	14.641 ± 0.842	14.629 ± 1.006	0.053	0.958
FIB, g/L	2.898 ± 0.345	2.841 ± 0.371	0.627	0.533
PLT, 10^9^/L	191.828 ± 48.717	177.156 ± 54.220	1.107	0.273

### 3.12 Comparison of coagulation and anemia-related indicators between the two subgroups

Regarding the coagulation-related indicators, there were no statistically significant differences in postoperative PT, TT, FIB, APTT, and PLT between the two subgroups (*p* > 0.05). In terms of anemia-related indicators on the postoperative 1st day, there were no significant differences in HB, RBC, and HCT between the two subgroups (*p* > 0.05). The detailed data were shown in Table 12.

**Table 12:** Comparison of postoperative coagulation and anemia-related indicators between the two groups.

Groups	Indobufen group (*n* = 18)	LMWH group (*n* = 11)	*t* value	*p*-value
APTT, s	28.156 ± 2.577	28.291 ± 1.789	−0.153	0.880
PT, s	11.561 ± 1.112	11.964 ± 0.530	−0.986	0.333
TT, s	14.414 ± 0.903	15.013 ± 0.596	−1.946	0.062
FIB, g/L	2.891 ± 0.384	2.910 ± 0.287	−0.140	0.889
PLT, 10^9^/L	192.110 ± 52.399	191.360 ± 44.469	0.039	0.969
HB, g/L	120.780 ± 14.036	112.090 ± 11.388	1.730	0.095
RBC, 10^12^/L	3.826 ± 0.415	3.594 ± 0.292	1.624	0.116
HCT, L/L	0.354 ± 0.041	0.330 ± 0.031	1.646	0.111

### 3.13 Dynamic changes and trends of anemia-related indicators between the two groups

**Figure 1:**
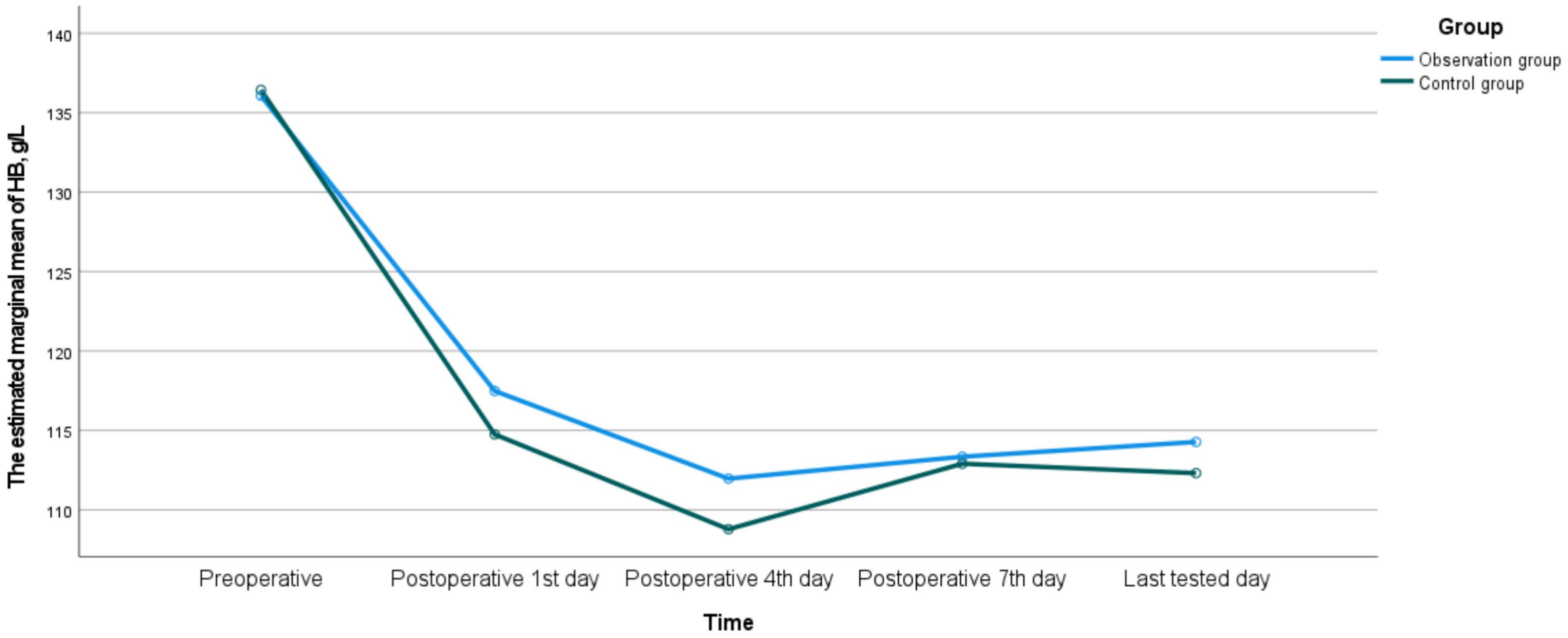
The dynamic changes of HB between the observation group and the control group.

**Figure 2:**
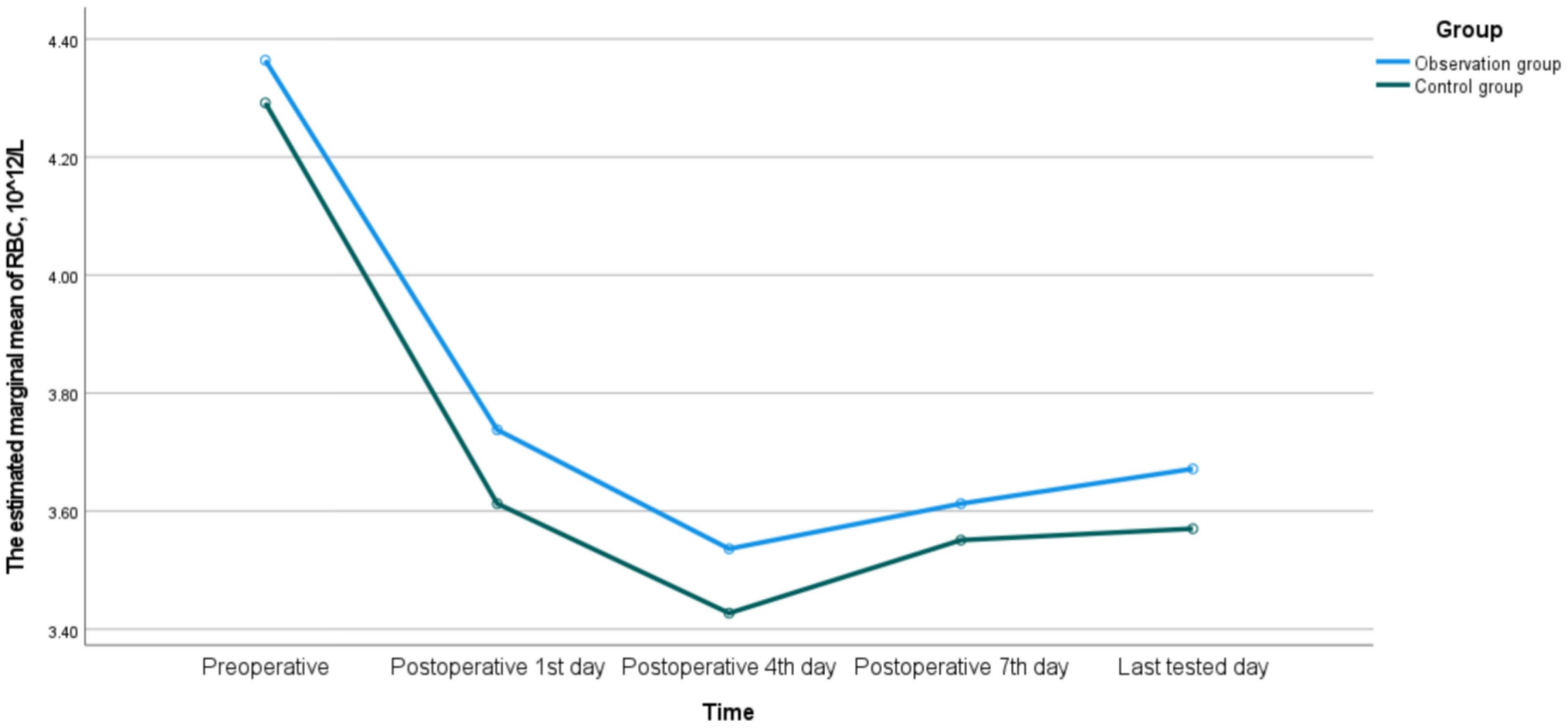
The dynamic changes of RBC between the observation group and the control group.

**Figure 3:**
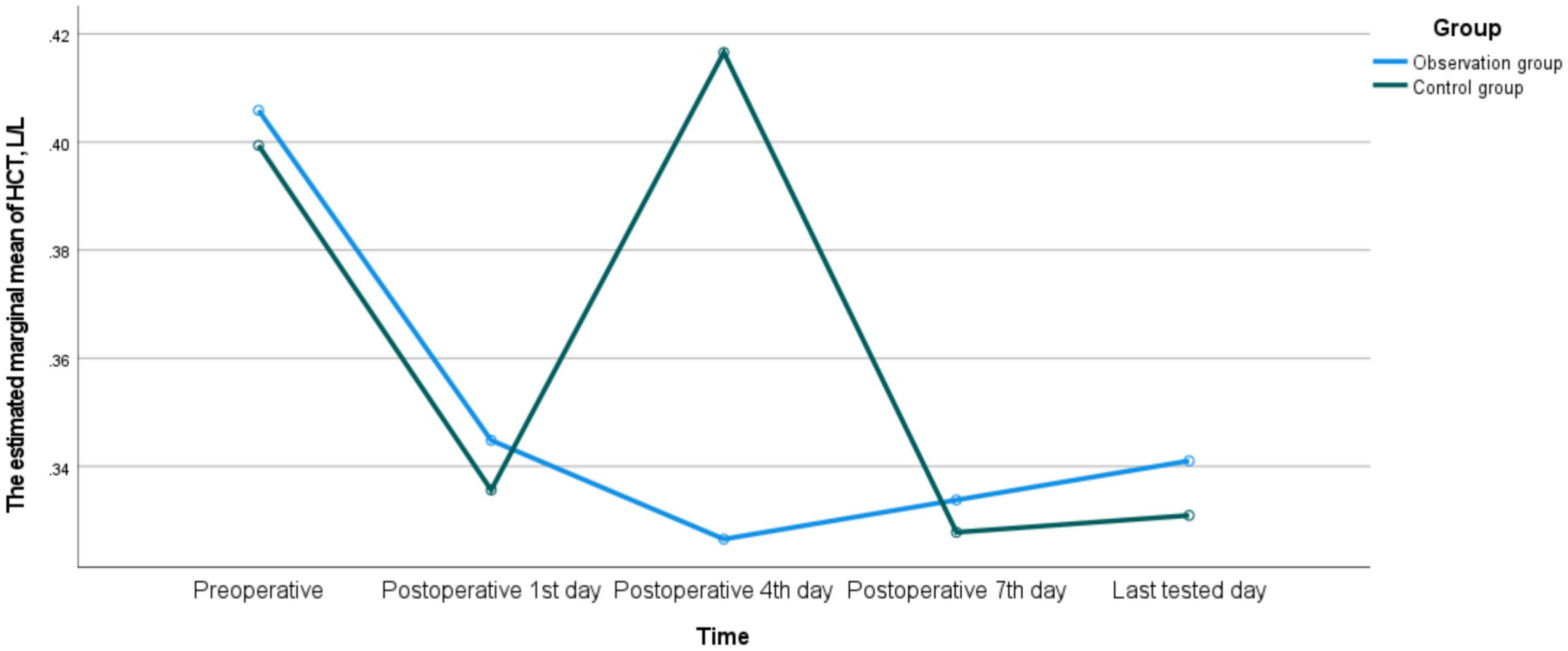
The dynamic changes of HCT between the observation group and the control group.

There were no significant differences in HB, RBC, and HCT between the two groups on the postoperative 1st, 4th, 7th, and last-tested days (*p* > 0.05). There was a time interaction in HB between the two groups, indicating that there were statistically significant differences in the monitoring values at different time points (*p* < 0.05), while there was no interaction between groups or Group * Time (*p* > 0.05), suggesting that the fluctuation trends of HB in the two groups were consistent. There was an interaction in RBC between time and Group * Time in the two groups (*p* < 0.05), indicating that there were statistically significant differences in the monitoring values at different time points (*p* < 0.05). Meanwhile, the fluctuation trends of RBC in the two groups were inconsistent. There was no interaction in HCT between the two groups, and the fluctuation trends of HCT in the two groups were consistent. The detailed data were shown in Table 13 and Figures 1–3.

**Table 13:** Comparison of the anemia-related indicators at different time points between the two groups.

Groups	Observation group (*n* = 29)	Control group (*n* = 32)	*t*/*χ*^2^/*Z*/*F* value	*p*-value	95% CI	
					Lower limit	Upper limit
HB, g/L						
Group			0.325	0.571		
Time			129.226	<0.001		
Time*Group			0.671	0.558		
Preoperative	136.069 ± 14.375	136.438 ± 10.163	−0.116	0.908	132.072	140.803
Postoperative 1st day	117.483 ± 13.577	114.750 ± 9.955	0.902	0.371	110.571	118.929
Postoperative 4th day	111.241 ± 11.122	109.438 ± 14.296	0.546	0.587	104.247	113.316
Postoperative 7th day	115.276 ± 12.623	111.156 ± 13.917	1.206	0.232	108.138	117.675
Last tested day	115.103 ± 12.639	111.563 ± 12.611	1.094	0.278	107.816	116.809
RBC, 10^12^/L						
Group			1.198	0.278		
Time			149.917	<0.001		
Time*Group			0.227	<0.001		
Preoperative	4.364 ± 0.416	4.292 ± 0.298	0.785	0.436	4.165	4.419
Postoperative 1st day	3.738 ± 0.385	3.613 ± 0.315	1.395	0.168	3.489	3.737
Postoperative 4th day	3.489 ± 0.355	3.470 ± 0.411	0.192	0.848	3.562	0.978
Postoperative 7th day	3.606 ± 0.384	3.557 ± 0.426	0.467	0.642	3.407	3.695
Last tested day	3.639 ± 0.430	3.600 ± 0.432	0.359	0.721	3.419	3.722
HCT, L/L						
Group			0.269	0.606		
Time			1.656	0.203		
Time*Group			0.860	0.359		
Preoperative	0.406 ± 0.040	0.399 ± 0.031	0.718	0.476	0.387	0.412
Postoperative 1st day	0.345 ± 0.039	0.336 ± 0.029	1.046	0.300	0.323	0.348
Postoperative 4th day	0.32 [0.30; 0.36]	0.32 [0.29; 0.35]	−1.334	0.182	0.271	0.562
Postoperative 7th day	0.336 ± 0.039	0.326 ± 0.039	0.929	0.357	0.314	0.342
Last tested day	0.340 ± 0.042	0.332 ± 0.037	0.730	0.469	0.317	0.345

## 4 Discussion

Under physiological conditions, the coagulation system and the anticoagulation system in the human body maintain a dynamic balance. The application of anticoagulant drugs may disrupt this balance and increase the risk of bleeding. In complex surgeries such as spinal surgery, the bleeding risk associated with perioperative anticoagulation therapy has always been a focus of attention. Currently, there is no consensus on whether anticoagulation treatment should be administered before PLIF (18), so it is not routinely given before surgery in clinical practice. However, for patients who have been on long-term anticoagulation treatment before admission, stopping the medication before surgery may disrupt the established balance and increase the risk of thromboembolism. In addition, studies on the impact of preoperative anticoagulation on bleeding and coagulation function in PLIF are still relatively limited.

This study retrospectively analyzed the effects of preoperative anticoagulant administration on postoperative coagulation function and blood loss in patients undergoing PLIF. The results showed that preoperative use of anticoagulant drugs did not significantly increase intraoperative blood loss, postoperative drainage volume, or the need for blood transfusion, and there were no significant differences in anemia-related indicators between the groups. In terms of coagulation function, only PT was significantly shortened, while TT, FIB, APTT, and PLT did not change significantly. In the subgroups of LMWH or indobufen, there were no significant differences in study related indicators on the first day after surgery. This again verified that the two anticoagulant drugs have similar efficacy. In the multivariate analysis, the results also indicated that preoperative anticoagulation was not a significant factor influencing blood loss and anemia. These results suggest that the preoperative application of specific anticoagulants, such as LMWH or indobufen, may be safe for patients undergoing PLIF.

Perioperative anticoagulants commonly used include unfractionated heparin, LMWH, factor Xa inhibitors, vitamin K antagonists, antiplatelet agents (19). Unfractionated heparin has gradually been replaced by LMWH because it requires regular monitoring of coagulation function and PLT (20). LMWH has a longer half-life, better bioavailability, and fewer adverse reactions such as bleeding, allergic reactions, and heparin-induced thrombocytopenia (21). In addition, it does not increase the risk of intraspinal hematoma formation that may lead to spinal cord compression (22). Rivaroxaban is a factor Xa inhibitor with anticoagulant effects similar to those of LMWH, but it is currently mainly used after hip and knee replacement arthroplasty (23, 24). Warfarin is a vitamin K antagonist with stable anticoagulant effects, but it requires regular monitoring of the international normalized ratio, which limits its perioperative applicability (25). Indobufen is an oral antiplatelet aggregation drug that reversibly inhibits platelet aggregation. Compared with aspirin, its inhibitory effect is reversed more quickly, with a lower risk of bleeding and overall better safety (26). Therefore, in this study, LMWH and indobufen tablets were used as alternative for preoperative anticoagulants.

Surgical safety is the prerequisite for all clinical research. This study focused on the safety of using anticoagulants before surgery. The results showed that all surgeries in the observation group were successfully completed without postoperative adverse reactions. In terms of coagulation indicators, there were no significant differences between the two groups in postoperative TT, APTT, FIB and PLT. However, the postoperative PT in the observation group was significantly shorter than that in the control group. One possible explanation is that PT is a sensitive indicator reflecting the extrinsic coagulation pathway. The reduction in PT may reflect the compensatory activation of the extrinsic coagulation pathway due to the release of a large amount of tissue factor caused by surgical stress and acute blood loss (27, 28). LMWH and indobufen tablets, respectively, exert their anticoagulant effects by inhibiting factor Xa through binding to antithrombin III and by selectively inhibiting the activity of cyclooxygenase-1 to prevent platelet aggregation, with a relatively small impact on PT (29, 30). Moreover, the reduction in PT did not lead to clinical adverse events such as DVT or postoperative hematoma, and APTT, TT, FIB and PLT remained stable, suggesting that preoperative anticoagulation did not affect the intrinsic and common coagulation pathways, and the overall coagulation function remained in a balanced state.

Theoretically, preoperative anticoagulation may put the body in a hypocoagulable state, thereby increasing intraoperative bleeding. Correspondingly, this may affect the progress of intraoperative operations and require additional intraoperative hemostatic measures. The ultimate possible outcome is prolonged surgical time, increased hemostasis difficulty and blood transfusion requirements, and possibly increased postoperative drainage volume. Therefore, there is still controversy over whether to use anticoagulant drugs before PLIF. One study showed that the application of anticoagulant drugs before double-segment PLIF did not increase intraoperative bleeding and blood transfusion (31). However, another study reported that preoperative anticoagulation for spinal fractures would lead to increased intraoperative bleeding and postoperative drainage (32). However, different findings were obtained in this study. Although the observation group had slightly higher surgical time, intraoperative blood loss, postoperative drainage volume and the number of blood transfusions than the control group, the differences were not statistically significant. This result may be related to the following factors. Firstly, surgical trauma-induced stress with the PLIF can activate the body’s powerful coagulation compensation system. The physiological regulation may partially counteract the inhibitory effect of anticoagulant drugs, and restore the balance between coagulation and anticoagulation. Secondly, as a mature and standardized surgical procedure, PLIF can better control intraoperative bleeding with the support of visualization technology, meticulous operation and effective hemostatic measures, without significantly prolonging the surgical time (33). Thirdly, the increase in blood loss in the observation group may not have reached the threshold for a statistically significant difference.

In terms of postoperative anemia indicators, although there were no significant differences in HB, RBC and HCT between the two groups, the trends of their changes were inconsistent. The fluctuation trends of HB and HCT were basically consistent, but the trend of RBC was different. The reasons for this phenomenon may be attributed to the following two aspects. Firstly, due to the influence of blood concentration and dilution effects, early postoperative fluid replacement, blood transfusion or third-space fluid transfer can lead to changes in plasma volume, which directly affects the concentrations of HCT and HB, but the impact on the absolute number of RBC is relatively indirect and delayed (34). Secondly, because the production and destruction of RBC are in a dynamic balance, postoperative stress may affect the lifespan of RBC and the compensatory production response of the bone marrow. These processes may not be completely synchronous in terms of time and magnitude for RBC, HB, and HCT (35). Meanwhile, the postoperative hospital stay was comparable between the two groups, further indicating that preoperative anticoagulation therapy did not significantly impact the postoperative recovery process. This might be attributed to the following three reasons. Firstly, the blood loss and blood transfusion conditions of the two groups were similar, so the degree of postoperative anemia was comparable. Additionally, the general conditions of the patients were equivalent, and the discharge criteria were the same. Secondly, active nutritional supplementation during the perioperative period might help correct the transient fluctuations in blood indicators. Finally, with the widespread implementation of PLIF, its perioperative management has become increasingly standardized and regulated (33).

To mitigate potential bias and adjust for potential confounding factors, a multivariate analysis was conducted on the factors that might affect blood loss-related indicators. The results indicated that the surgical segment was a significant factor influencing the indicators, while some other potential factors such as hypertension, disease type, and preoperative use of anticoagulant drugs were not. This was partially different from the conclusion of the comparison of general data between the two groups in this study. The possible reason is that different statistical methods have different efficacies. Concurrently, it also suggests that for multi-segment surgeries, more comprehensive perioperative blood management is necessary in clinical practice.

This study had several limitations. Firstly, it was a small-sample retrospective study, which may have selection bias. Future studies with larger samples, multiple centers, and a prospective design are needed for verification. Secondly, blood loss includes both overt and hidden blood loss. This study only discussed overt blood loss in the analysis, which may affect the comprehensiveness of the conclusion. Finally, this study only observed conventional coagulation indicators and failed to comprehensively and systematically evaluate other coagulation-related molecular markers.

## 5 Conclusion

The results of this study indicate that the use of anticoagulants before PLIF has no significant effect on postoperative coagulation function indicators (except for PT), including TT, FIB, APTT, and PLT, and has no significant impact on intraoperative and postoperative blood loss. However, this study is a single-center retrospective study, and the reliability of the conclusion still needs to be further verified by large-sample, multi-center prospective studies.

## Data Availability

The original contributions presented in the study are included in the article/Supplementary material, further inquiries can be directed to the corresponding author.
